# Study on the cost attributable to central venous catheter-related bloodstream infection and its influencing factors in a tertiary hospital in China

**DOI:** 10.1186/s12955-018-1027-3

**Published:** 2018-10-11

**Authors:** Yuanyi Cai, Min Zhu, Wei Sun, Xiaohong Cao, Huazhang Wu

**Affiliations:** 1 Department of Health Service Management, School of Humanities and Social Sciences, China Medical University, No.77 Puhe Road, Shenyang North New Area, Shenyang, Liaoning Province 110122 People’s Republic of China; 2 Department of Social Medicine, School of Public Health, China Medical University, No.77 Puhe Road, Shenyang North New Area, Shenyang, Liaoning Province 110122 People’s Republic of China

**Keywords:** Catheter–related bloodstream infection, Cost, Influencing factor

## Abstract

**Background:**

Central venous catheters (CVC) have been widely used for patients with severe conditions. However, they increase the risk of catheter-related bloodstream infection (CRBSI), which is associated with high economic burden. Until now, no study has focused on the cost attributable to CRBSI in China, and data on its economic burden are unavailable. The aim of this study was to assess the cost attributable to CRBSI and its influencing factors.

**Methods:**

A retrospective matched case-control study and multivariate analysis were conducted in a tertiary hospital, with 94 patients (age ≥ 18 years old) from January 2011 to November 2015. Patients with CRBSI were matched to those without CRBSI by age, principal diagnosis, and history of surgery. The difference in cost between the case group and control group during the hospitalization was calculated as the cost attributable to CRBSI, which included the total cost and five specific cost categories: drug, diagnostic imaging, laboratory testing, health care technical services, and medical material. The relation between the total cost attributable to CRBSI and its influencing factors such as demographic characteristics, diagnosis and treatment, and pathogenic microorganism, was analysed with a general linear model (GLM).

**Results:**

The total cost attributable to CRBSI was $3528.6, and the costs of specific categories including drugs, diagnostic imaging, laboratory testing, health care technical services, and medical material, were $2556.4, $112.1, $321.7, $268.7, $276.5, respectively. GLM analysis indicated that the total cost was associated with the intensive care unit (ICU), pathogenic microorganism, age, and catheter number, according to the sequence of standardized estimate (β). ICU contributed the most to the model R-square.

**Conclusion:**

Central venous catheter–related bloodstream infection represents a great economic burden for patients. More attentions should be paid to further prevent and control this infection in China.

## Background

Central venous catheters (CVC) have been widely used in the treatment of patients with severe diseases. However, the utilization of CVC may cause catheter-related bloodstream infection (CRBSI), which is a great economic burden on the patients. In recent years, many studies have been carried out worldwide to focus on the cost of CRBSI. According to our knowledge, four designs including a retrospective matched case-control study [1–3], prospective research [4–8], multivariate analysis [9], and a case report [10], have been used to evaluate the cost attributable to CRBSI. The cost of CRBSI has been estimated to be between $11,971and $69,332 in America [1, 4, 5, 9, 10]. Studies performed in European countries have reported the cost to be in the ranges of €13,585 to €29,909 [2, 6]. The value calculated in developing countries has ranged between $4888 and $11,591 [7, 8]. A recent Japanese study reported an excess cost of $57,090 for CRBSI [3].

In China, CRBSI is currently one of the four nosocomial infections monitored by the Chinese government [11], and it has aroused increasing public attention. A prospective investigation of CRBSI was conducted across 12 provinces and showed that the incidence rate was between 0.69 and 14.22 per 1000 central catheter days (133 out of 4256 patients infected by CRBSI in 55 intensive care units (ICU) from 41 hospitals monitored from October 2013 to March 2014) [12]. However, there is still no report about the cost attributable to CRBSI in China. The factors influencing this economic burden are also unknown. The Chinese government is currently trying to solve the problem of the high costs of obtaining medical services with new medical reform. Distinguishing between the cost of primary disease and that of CRBSI can help the government to determine the origin of the economic burden and further evaluate the burden from CRBSI itself, which would be useful to establish targeted control strategies. Furthermore, an analysis of factors that influence the cost attributable to CRBSI will also provide evidence to hospitals for improving medical quality and to patients for exerting better self-care during hospitalization.

The purpose of the present study was to assess the cost attributable to CRBSI and explore its influencing factors for the government, hospitals and patients. Because the incidence of CRBSI is low in China [12], and the information in Chinese hospital medical records is not as sufficient as that in developed countries, methods such as prospective research, multivariate analysis, and case reports were not feasible. Thus, this study was a retrospective matched case-control study. The cost attributable to CRBSI and its influencing factors available in the hospital information system (HIS) and medical records, including demographic characteristics, diagnosis and treatment, and pathogenic microorganisms, were analysed. We believe our findings can help the government, hospitals and patients to prevent and control this infection.

## Methods

### Definition

CRBSI is described as the presence of bacteraemia or fungemia in patients with a catheter or within 48 h after the catheter removal without the presence of any other source of infection. The clinical signs of patients with CRBSI are fever (> 38 °C), chills, and hypotension. Laboratory microbiology examinations have shown a positive result for the peripheral blood sample culture or the catheter tip culture of the same microorganism [13].

### Study population

The study was conducted in a tertiary hospital affiliated with China Medical University. It is a public 3346-bed hospital. All patients (age ≥ 18 years old) with central venous catheter-related bloodstream infection in every department from January 2011 to November 2015 were included, and there were a total of 94 patients for the case group. Meanwhile, a matched (1:1) case-control study design was utilized. The controls were defined as patients with CVC insertion but without CRBSI. A total of three key variables were used in the matching procedure, including age (± 10 yr.), principal diagnosis, and history of surgery [8, 14].

### Assessment of cost attributable to CRBSI

The cost data were derived from the patients’ true hospital expenditure and were provided by the financial department of the hospital. The duration from the time of admission of the patient to the day of discharge was selected. The costs of patients with CRBSI were analysed and compared with those of the patients without CRBSI. The difference in cost between the two groups during the hospitalization was calculated as the cost attributable to CRBSI. This difference included the total cost and five specific cost categories: 1) drug, 2) diagnostic imaging, 3) laboratory testing, 4) health care technical service, and 5) medical material. The analysis of the drug included only western medicine. Diagnostic imaging included the diagnosis of Doppler ultrasonography, electrocardiogram, computed tomography (CT), and other small medical devices. Laboratory testing mainly included a routine blood test, and a bacterial culture. Health care technical service included surgery, physical examination, nursing, medical disposition, and group consultation. Medical material was defined as the medical consumables and equipment used in the hospital such as catheters and sutures. The currency exchange rate used was US$1 to ¥ 6.8977.

### Measurements of demographic characteristics, diagnosis and treatment, and pathogenic microorganism

Demographic characteristics included age, sex, occupation, and immunosuppressive drug history. Occupation was categorized as worker, farmer, office clerk, retiree, and others according to the information provided by the patients on the front page of the medical record.

Diagnosis and treatment was assessed based on 5 items: 1) ICU, 2) principal diagnosis, 3) other diagnoses, 4) operation level, and 5) catheter number. If the patients were admitted to the ICU during the hospital stay, this indicated that they had a severe condition and that their treatment would cost much more than those of other patients. The principal diagnosis was categorized as cancer, inflammation, and others. Patients with cancer and severe inflammation made an impact on the treatment of CRBSI. Other diagnoses were marked “yes” if diagnoses such as hypertension and diabetes were found in the patients’ medical records. The operation level was categorized as not performing an operation or performing an operation and ranged from level one to level four based on the complexity of the surgery [15], with level four representing the most difficult operation. The catheter number was assessed through the total number of CVC, tracheal cannula, urinary catheter, drainage tube, and stomach tube. Patients with few (less than or equal to four) catheters were combined with patients with five catheters into “≤ 5 group”.

Pathogenic microorganisms were categorized as gram-positive bacteria (G+), gram-negative bacteria (G-), and fungi to explore their impact on the cost.

### Statistical analysis

The difference in age between the two groups after the matching procedure was evaluated by Student’s t-test. The difference in cost for the patients in the case and control group and the different influencing factors in the univariate analysis were tested by the Wilcoxon rank sum test. General linear model analysis was used to clarify the influencing factors for the total cost attributable to CRBSI. Variables significant at the 0.25 level in the univariate analysis were entered in the model. Items with *P* > 0.15 were eliminated one at a time in the sequence of *P* value. When a variable was eliminated, it remained in the model as a confounder if any remaining parameter estimate changed over 20%. In the study, no confounder was found during elimination. SAS for Windows (V8.2) was used for the statistical analyses.

## Results

### Fundamental results of matching

According to the matching criteria, 81 patients were successfully matched with control patients (the information of the other 13 patients could not be retrieved through the HIS). The average age of the case group was 62.2 ± 11.2 years old, and that of the control group was 61.9 ± 11.3 years old (*P* = 0.867). Twenty-five types of principal diagnosis were involved. Pancreatic tumor (14 pairs), rectal cancer (10 pairs), cholangiocarcinoma (9 pairs), gastric cancer (8 pairs), and liver cancer (5 pairs) were the main primary diseases, and made up 56.8% of all pairs. Considering the history of surgery, twenty-one types of operations associated with the primary diseases were performed.

### Evaluation of the cost attributable to CRBSI

The median cost in the CRBSI group was $12,515.2, which was significantly higher (*P* <  0.001) than that in the control group. The total median cost attributable to CRBSI was $3528.6. Furthermore, the differences between the drug costs, diagnostic imaging costs, laboratory testing costs, technical fees, and medical material costs of the two groups were $2556.4, $112.1, $321.7, $268.7, and $276.5, respectively (Table 1).

**Table 1:** Medical costs attributable to CRBSI ($)

	Median cost in the CRBSI group	Median cost in the control group	Medical cost attributable to CRBSI	*P*
Total	12,515.2	8450.5	3528.6	< 0.001
Drug	6576.0	4056.9	2556.4	< 0.001
Diagnostic imaging	401.3	328.8	112.1	< 0.001
Laboratory testing	906.1	536.0	321.7	< 0.001
Technical fees	1325.7	987.9	268.7	< 0.001
Medical material	1697.3	1369.4	276.5	0.002

### Influencing factors of the cost attributable to CRBSI

The characteristics and distributions of the total cost attributable to CRBSI are shown in Table 2. There were significant differences in the ICU factors (*P* <  0.001) and catheter numbers (*P* = 0.002) of the patients. In addition, there was a mild correlation between the total cost and other diagnoses (*P* = 0.053).

**Table 2:** The characteristics and distributions of the total cost attributable to CRBSI

Variables		n	Median($)	*P*
Age(yr.)	≤ 59	33	2977.9	0.152
	> 60	48	4424.3	
Sex	Male	50	3070.5	0.290
	Female	31	4042.8	
Occupation	Worker	7	1902.5	0.702
	Farmer	17	2977.9	
	Office clerk	9	3528.6	
	Retiree	30	4436.9	
	Others	18	3495.1	
Immunosuppressive drug history	No	69	3458.8	0.481
	Yes	12	5316.0	
ICU	No	65	2420.7	< 0.001
	Yes	16	9154.3	
Principal diagnosis	Cancer	59	3087.0	0.218
	Inflammation	6	8760.0	
	Others	16	4368.9	
Other diagnoses	No	28	1930.6	0.053
	Yes	53	4042.8	
Operation level	≤ 2	24	2920.2	0.228
	3	24	6002.2	
	4	33	3087.0	
Catheter number	≤ 5	64	2549.0	0.002
	6	17	7587.4	
Pathogenic microorganism	G-	25	2335.6	0.137
	G+	42	3601.9	
	Fungus	14	6340.1	

The results of the general linear model analysis for clarifying the factors influencing the economic burden attributable to CRBSI are shown in Tables 3 and 4. The additional cost was significantly related to ICU, pathogenic microorganism, age, and catheter number according to the sequence of the standardized estimate (β). The contribution to the model R-square attributable to the four factors (ΔR-square) was 0.1488, 0.0326, 0.0339, and 0.1078, respectively.

**Table 3:** The general linear model analysis for clarifying the major factors influencing the economic burden attributable to CRBSI

Variables	Parameter estimate (B)	Standardized estimate (β)	Model R-Square
Intercept	3997.14		
ICU (Yes vs. No)	4962.37^***^	0.40	
Pathogenic microorganism (G+ vs. fungus)	− 3315.04^**^	−0.31	
Pathogenic microorganism (G- vs. fungus)	− 2246.35^*^	− 0.23	
Age (yr.) (> 60 vs. ≤ 59)	2154.24^**^	0.21	
Catheter number (6 vs. ≤ 5)	2196.67^*^	0.18	0.3231^***^

^*^P < 0.15. ^**^P < 0.05. ^***^P < 0.01

**Table 4:** The contributions of factors of the model R-square

	Demographic characteristics	Diagnosis and treatment		Pathogenic microorganism
Statistic				
	Age			
		Catheter number		
			ICU	
				Pathogenic microorganism
F	3.81	7.6^**^	11.92^**^	8.64^**^
R^2^	0.0339	0.1417	0.2905	0.3231
ΔR^2^	0.0339	0.1078	0.1488	0.0326

^**^P < 0.01

## Discussion

This study is the first to report the cost attributable to CRBSI and its influencing factors in China. Our results revealed that the median cost attributable to CRBSI was $3528.6 and was only lower than those of the five most costly diseases, which were myocardial infarction-coronary artery bypass grafting ($9700.9), acute myocardial infarction ($4901.9), gastric cancer ($4101.2), lung cancer ($3785.1), and esophageal cancer ($3601.9) according to the list of 30 diseases whose economic burden was evaluated by the National Health Commission [16]; however, the cost attributable to CRBSI was more than that of the other diseases.

In addition, the cost attributable to CRBSI was 2.3 times that of the per capita net income ($1561.7) of the farmers and made up 78.0% of the disposable income of the urban residents ($4522.5) in China [17]. Therefore, the occurrence of CRBSI further increased the economic burden for the patients, especially for those from rural areas or with limited insurance coverage and reimbursement.

The cost attributable to CRBSI in this study was significantly lower than the level of developed countries and slightly lower than that of developing countries. Three reasons contribute to the differences. First, unlike the studies in other countries, our study showed that most patients in this sample hospital were from general wards (80.2%) rather than from ICUs, which decreased the cost to some extent. Second, there are significant differences in economic development and medical price across the countries, which may impact the cost. Third, the unsound health care pricing system in China is another reason. The value of the medical staff is greatly underestimated. In the specific cost categories, our study revealed that the median drug cost was the main component. However, the median health care technical fee, which reflected the value of the medical staff, was underestimated and only made up 7.6% of the total additional cost. The unsound pricing system is one of the structural problems in the health care system in China and needs to be changed with the new medical reform.

The influencing factors of age, catheter number, ICU, and pathogenic microorganisms were found to be crucial. Moreover, the ICU was found to have the strongest association with the cost caused by CRBSI among all four factors, which made up the major contribution (0.1488) to the model R-Square. In the ICU, patients with severe situations need special care, sophisticated medical monitoring devices, and better drugs, all of which lead to higher expenditure for the treatment of CRBSI. Furthermore, the price elasticity of demand is lower in the ICU because the patients and relatives are faced with extremely limited options concerning life and health [18]. The number of catheters was another important factor influencing the cost attributable to CRBSI. Other kinds of catheters were used such as tracheal cannula, urinary catheter, drainage tube, and stomach tube, and other types of infections might occur, which makes the recovery of CRBSI much more difficult [19].

Both age and pathogenic microorganisms, contributed less to the model R-Square. Unlike those of young patients, the organ function and immune system of old patients decline, which makes treatment more difficult [20]. China is becoming an ageing society. It is clear that the health care expenditures of the whole nation will be significantly increased [21] if CRBSI cannot be prevented in elderly patients. Comparing the pathogenic microorganisms, fungal infections had a much higher impact on cost than G+ and G- bacterial infections. The result was consistent with the study of Itaru Nakamura, who reported that Candida CVC-CRBSI incurred the highest cost. This was likely due to the broader consequences of bloodstream fungal infection such as severe sepsis and multiple organ failure [3].

There are two limitations in the study. First, the lower incidence of CRBSI in China resulted in a small sample size in our study, which had an impact on the cost results and influencing factor analysis. Second, for the matched case-control study, the most serious problem was choosing the matching criteria. There is no doubt that the more criteria are chose, the more accurate the result will be. However, if the matching criteria are too stringent, it is difficult to find control patients and further decrease the sample size [22]. Therefore, three core matching variables were chose in our study to maintain the balance between accuracy and maximum sample size.

## Conclusions

The present study assessed the cost attributable to CRBSI and explored its influencing factors. Our findings revealed that CRBSI introduced a heavy economic burden for patients, especially older patients, those with severe conditions (ICU or more catheters), and those infected by fungi. Although the incidence of CRBSI in China has been lower in recent years through measures taken by the government and hospitals, more attentions should be paid to further prevent and control the infection; if possible, the level of medical insurance payment for the CRBSI patients should be increased during the new medical reform, with the purpose of relieving the economic burden among patients in China.

## Abbreviations

CRBSI: Catheter-related bloodstream infection
CT: Computed tomography
CVC: Central venous catheter
G +: Gram-positive bacterium
G-: Gram-negative bacterium
GLM: General linear model
HIS: Hospital information system
ICU: Intensive care unit
